# Implementing a school-based comprehensive sexual health education intervention in Italy: experiences from the field

**DOI:** 10.1093/eurpub/ckac129.742

**Published:** 2022-10-25

**Authors:** D Martinelli, R Galipò, P Meli, M Farinella, A Chinelli, AT Palamara, B Suligoi, M Oldrini, L Colaprico, L Tavoschi

**Affiliations:** 1 Università di Foggia, Foggia, Italy; 2 Associazione Nazionale per la Lotta control', Rome, Italy; 3 Coordinamento Italiano Case Alloggio/AIDS, Bergamo, Italy; 4 Circolo di Cultura Omosessuale Mario Mieli, Rome, Italy; 5 Università di Pisa, Pisa, Italy; 6 Università La Sapienza, Rome, Italy; 7 National Institute of Health, Rome, Italy; 8 Lega Italiana Lotta all'AIDS, Rome, Italy; 9 Croce Rossa Italiana, Rome, Italy

## Abstract

**Introduction:**

This study describes the preliminary results of a School-based sexuality education (SBSE) pilot activity developed and implemented within EduForIST project, funded by the Italian Ministry of Health.

**Methods:**

The pilot activity (5 modules of 2 hours each delivered per classroom) targeted lower secondary schools students. A total of 20 schools located in 4 different Italian regions participated. The educators were staff of several HIV/AIDS civil society organisations operating in Italy. A 2-days intensive workshop for educators was performed. Pre and post tests were conducted.

**Results:**

At the time of submission, pre-test results were available from 14 classrooms of 5 schools within 2 Italian Regions, for a total of 266 students. Among these, 37,4% were unsure that personal identity is built through social comparison; 21,8% reported that emotions don't get more intense during adolescence, while 18,1% were unsure about the response; 42,1% reported a higher level of uncertainty concerning the definitions of gender identity, sexual orientation and stereotype. The highest level of uncertainty were reported for STIs symptoms (58,7%), impact of treatment on HIV+ people (61,9%) and efficacy of contraceptive pills in preventing STIs (43,4%). The post-test results were available for 153 students. Pre/post analysis showed an increase of correct answers (p < 0.05) for 12 of 15 items investigated. A total of 102 students responded to the satisfaction questionnaire, with preliminary positive results.

**Conclusions:**

Since activities are ongoing, further data will be soon available for more exhaustive analyses. Early pre/post evaluations suggested that the pilot experience was effective in enhancing knowledge and decreasing uncertainty in the different domains addressed in the pilot. Evidence collected through this study shall raise awareness among decision makers on the urgency of introducing CSE in Italian school curricula and inform future policy options.

